# Docking prediction, molecular dynamics and antibiofilm effect of linalool against *Candida albicans* strains from vulvovaginal secretions

**DOI:** 10.7705/biomedica.7709

**Published:** 2026-04-28

**Authors:** Cássio Ilan Medeiros, Rawny Galdino Gouveia, Victor Targino Gomes, Gislaine Silva, Ulrich Vasconcelos Gomes, Abrahão Alves Filho, Edeltrudes de Oliveira

**Affiliations:** 1 Departamento de Ciências Farmacêuticas, Universidade Federal da Paraíba, João Pessoa, PB, Brasil Universidade Federal da Paraíba Departamento de Ciências Farmacêuticas Universidade Federal da Paraíba João Pessoa PB Brasil; 2 Departamento de Biotecnologia, Universidade Federal da Paraíba, João Pessoa, PB, Brasil Universidade Federal da Paraíba Departamento de Biotecnologia Universidade Federal da Paraíba João Pessoa PB Brasil; 3 Departamento de Microbiología, Universidade Estadual de Londrina, Londrina, PR, Brasil Universidade Estadual de Londrina Departamento de Microbiología Universidade Estadual de Londrina Londrina PR Brasil; 4 Unidade Acadêmica de Ciências Biológicas, Universidade Federal de Campina Grande, Polo: Patos, PB, Brasil Universidade Federal de Campina Grande Unidade Acadêmica de Ciências Biológicas Universidade Federal de Campina Grande Polo: Patos PB Brasil

**Keywords:** Candida albicans, biofilms, monoterpenes, antifungal agents, candidiasis, vulvovaginal., Candida albicans, biopelículas, monoterpenos, antifúngicos, candidiasis vulvovaginal.

## Abstract

**Introduction.:**

Vulvovaginal candidiasis is a prevalent fungal infection, predominantly caused by *Candida albicans*. Biofilm formation plays a central role in disease persistence and therapeutic failure by enhancing fungal tolerance to conventional antifungal agents.

**Objective.:**

To evaluate the antibiofilm activity of linalool against *C. albicans* strains isolated from vulvovaginal secretions and to investigate its potential molecular interactions with 1,3-β-glucano synthase, a key enzyme involved in fungal cell wall biosynthesis.

**Materials and methods.:**

The antibiofilm effect of linalool was assessed *in vitro* using the crystal violet assay against a clinical isolate (*C. albicans* LM 129) and a reference strain (*C. albicans* ATCC 76485). Nystatin was included as a comparator antifungal. In parallel, molecular docking analyses were performed using AutoDock 4.2, followed by molecular dynamics simulations conducted in GROMACS 2022.3 to evaluate the stability of the linalool-enzyme complex.

**Results.:**

Linalool significantly reduced biofilm formation in both C. *albicans* strains, with an average inhibition of 74.65%, whereas nystatin exhibited limited antibiofilm activity. Docking studies revealed that linalool interacts with the active site of 1,3-β-glucano synthase, presenting a binding free energy (*ΔG*) of -6.0 kcal/mol, comparable to the control ligand castanospermine (-7.0 kcal/mol). Molecular dynamics simulations demonstrated structural stability of the linalool-enzyme complex over a 30 ns trajectory, supporting the persistence of these interactions under dynamic conditions.

**Conclusions.:**

Linalool exhibits relevant antibiofilm activity against C. *albicans* and shows stable interactions with 1,3-β-glucano synthase, suggesting a plausible inhibitory mechanism. These findings support the potential of linalool as a natural scaffold for the development of alternative therapeutic strategies for vulvovaginal candidiasis.

## Resumen

**Introducción.:**

La candidiasis vulvovaginal es una infección fúngica frecuente causada principalmente por *Candida albicans.* La formación de biopelículas constituye un factor clave en la persistencia de la infección y en la disminución de la eficacia de los tratamientos antifúngicos convencionales.

**Objetivo.:**

Evaluar la actividad anti-biopelícula del linalol frente a cepas de C. albicans aisladas de secreciones vulvovaginales e investigar sus posibles interacciones moleculares con la enzima 1,3-β-glucano sintasa, esencial para la biosíntesis de la pared celular fúngica.

**Materiales y métodos.:**

La actividad anti-biopelícula del linalol se evaluó mediante pruebas *in vitro* con cristal violeta frente a una cepa clínica (*C. albicans* LM 129) y una cepa de referencia (*C. albicans* ATCC 76485). La nistatina se utilizó como antifúngico comparador. Además, se realizaron estudios de acoplamiento molecular utilizando AutoDock 4.2, seguidos de simulaciones de dinámica molecular en GROMACS 2022.3 para analizar la estabilidad del complejo ligando-enzima.

**Resultados.:**

El linalol redujo significativamente la formación de biopelículas en ambas cepas de *C. albicans*, con una inhibición promedio del 74,65 %, mientras que la nistatina mostró una actividad limitada. Los estudios de acoplamiento indicaron que el linalol interactúa con el sitio activo de la 1,3-β-glucano sintasa, presentando una energía libre de unión (*ΔG*) de -6,0 kcal/mol, comparable a la del ligando de control castanospermine (-7,0 kcal/mol). Las simulaciones de dinámica molecular confirmaron la estabilidad estructural del complejo durante 30 ns.

**Conclusiones.:**

El linalol presenta una actividad anti-biopelícula relevante frente a *C. albicans* y establece interacciones estables con la enzima 1,3-β-glucano sintasa, lo que sugiere un posible mecanismo inhibitorio. Estos resultados respaldan su potencial como compuesto natural para el desarrollo de estrategias terapéuticas alternativas contra la candidiasis vulvovaginal.

Vulvovaginal candidiasis is one of the most common fungal infections affecting women of reproductive age, with *Candida albicans* accounting for most cases ^1^. Although azoles and polyenes remain the mainstay of therapy, recurrence and therapeutic failure are frequently reported, highlighting the limitations of current antifungal regimens ^2^. These clinical challenges are often associated with the ability of *C. albicans* to form biofilms on mucosal surfaces, a process that enhances fungal persistence and markedly reduces susceptibility to antifungal agents ^3^.

Biofilms are structured microbial communities embedded in a self-produced extracellular matrix that confers mechanical protection and promotes phenotypic heterogeneity. In *C. albicans*, biofilm formation is a multifactorial process regulated by morphogenetic transitions, adhesion molecules, and cell wall remodeling enzymes ^4^. Cells within biofilms exhibit increased tolerance to antifungal drugs and host immune responses, contributing to chronic and recurrent infections. Consequently, the identification of compounds capable of preventing or disrupting fungal biofilms has become a priority in antifungal research ^5^.

The fungal cell wall plays a pivotal role in biofilm development and maintenance, serving as both a structural scaffold and a dynamic interface with the host environment ^6^. Among its components, β-glucans are essential polysaccharides that provide rigidity and integrity to the cell wall. The enzyme β-1,3-glucan synthase catalyzes the synthesis of β-1,3-glucans and represents a validated antifungal target, as evidenced by the clinical efficacy of echinocandins ^7^. However, the emergence of resistance and adverse effects associated with existing drugs underscore the need for alternative or complementary therapeutic approaches ^8^.

Natural products have long been recognized as valuable sources of bioactive molecules with antimicrobial properties. Among them, monoterpenes such as linalool have demonstrated antifungal activity against *Candida* species, although their effects on biofilm formation and underlying molecular mechanisms remain incompletely understood ^9^.

In this context, the present study aimed to investigate the antibiofilm activity of linalool against *C. albicans* strains isolated from vulvovaginal secretions and to explore its potential molecular interactions with 1,3-β-glucan synthase through molecular docking and molecular dynamics simulations.

## Materials and methods

### Substances and microorganisms

Linalool (LIN; 3,7-dimethyl-1,6-octadien-3-ol) and nystatin were used as test substances. Linalool (purity ≥98.7%) was obtained from Quinarí™ (Ponta Grossa, PR, Brazil), while nystatin was purchased from Sigma-Aldrich™ (São Paulo, SP, Brazil). Stock solutions of linalool were freshly prepared immediately prior to each assay due to its volatile nature. The compound was initially solubilized in dimethyl sulfoxide (3%) and polysorbate 80 (1%), followed by dilution with sterile distilled water to obtain a final volume of 80 ml. This procedure resulted in a homogeneous emulsion corresponding to the previously determined minimum inhibitory concentration of 64 µg/ml ^10^.

The clinical strain *C. albicans* LM 129, isolated from vulvovaginal secretions and previously characterized as fluconazole-resistant, was obtained from the fungal collection of the Laboratory for Research on Antibacterial and Antifungal Activity of Natural or Synthetic Bioactive Products of the Federal University of Paraíba (Brazil). In addition, *C. albicans* ATCC 76485 (American Type Culture Collection) was included as a reference strain.

For all *in vitro* assays, fungal inocula were prepared from fresh cultures suspended in 0.85% saline solution, with cell density adjusted to 0.5 McFarland standard, corresponding to approximately 1-5x10^6^ colonyforming units per milliliter (CFU/ml) ^11^.

### Biofilm formation test

The inhibitory effect of linalool on *C. albicans* biofilm formation was assessed using the crystal violet staining method (NewProv, Pinhais, PR, Brazil). Briefly, sterile polystyrene microtubes (1.5 ml) were filled with Roswell Park Memorial Institute (RPMI)-1640 medium (800 µl), followed by the addition of 100 µl of linalool emulsion at its previously determined minimum inhibitory concentration (64 µg/ml), and 100 µl of standardized fungal suspensions of both *C. albicans* LM 129 and ATCC 76485.

For comparison, the same experimental design was applied using nystatin at its minimum inhibitory concentration (4 µg/ml), selected due to its fungicidal activity against the LM 129 strain.

The microtubes were incubated at 35 ± 2 °C for 48 h under static conditions to allow biofilm development. After incubation, the culture medium was carefully removed, and non-adherent cells were eliminated by washing the tube walls three to five times with distilled water. The microtubes were then air-dried at room temperature for 1 h. Subsequently, the adherent biofilms were stained with 1% crystal violet solution (1000 µl) for 20 min. Excess dye was removed by gentle washing with distilled water, and the bound stain was solubilized by adding 1000 µl of 95% ethanol (Química Moderna, Barueri, SP, Brazil).

The absorbance of the resulting crystal violet-ethanol solution was measured at 590 nm using a spectrophotometer (Kasvi™, 4NM). Negative controls consisted of microtubes containing RPMI-1640 medium supplemented only with the solvent system (dimethyl sulfoxide and Tween 80), whereas positive controls included fungal suspensions incubated in RPMI-1640 without antifungal agents. All experiments were performed in triplicate.

The percentage of biofilm inhibition was calculated according to the formula [(ODC - ODT) / ODC ^x^ 100], where ODC represents the mean optical density of the untreated control and ODT corresponds to the mean optical density obtained for treated samples ^12^.

Based on adhesion values, biofilms were classified as weak (<40%), moderate (40.01-79.99%), or strong (>80%) ^13^. Likewise, the degree of inhibition of biofilm formation was categorized as weak (<40%), moderate (40.01-79.99%), or strong (>80%) ^13^.

### Molecular docking

Molecular docking simulations were carried out to investigate the interaction between linalool and the enzyme 1,3-β-glucan synthase, a key protein involved in *C. albicans* cell wall biosynthesis. The three dimensional structure of the enzyme (Protein Data Bank ID: 1EQC) was retrieved from the Protein Data Bank and prepared using PyMOL™, version 2.5.3. All crystallographic water molecules and the co-crystallized ligand castanospermine were removed prior to the simulations ^14^.

The chemical structure of linalool was obtained from the PubChem database and subjected to geometric optimization using Avogadro™, version 1.2.0. Energy minimization was subsequently performed at physiological pH 7.4 using MOPAC2012 at the PM7 semi-empirical level, applying the MMFF94 force field to ensure structural stability of the ligand.

Protein preparation for docking was performed using AutoDock Tools™, version 1.5.6, including the addition of polar hydrogens, assignment of «oilman partial charges, and merging of non-polar hydrogens.

The docking grid was defined to encompass the predicted active site region of the enzyme, considering the flexibility of key amino acid residues (Glu27, Tyr29, His135, Asn146, Asn191, Glu192, Tyr255, Glu292, and Trp363). The grid box was centered at coordinates (34.475, 36.263, 55.769 Å), with dimensions of 40 × 40 × 40 Å and a grid spacing of 0.375 Å.

Docking calculations were performed using AutoDock™, version 4.2, employing the Lamarckian genetic algorithm with 100 independent runs under default parameters. Binding affinity was evaluated based on the estimated free energy of binding (*ΔG*) and inhibition constant (K_i_ ), and the most favorable binding conformations were selected according to the lowest ΔG values ^15^.

The resulting ligand-protein complexes were analyzed using PyMOL™, version 2.5.3, and Discovery Studio 2021 to identify binding modes, interacting residues, and the nature of intermolecular interactions. Methodological validation was conducted through molecular redocking of the native ligand, and the docking protocol was considered reliable when the root- mean-square deviation between the experimental and predicted poses was lower than 2.0 Å ^16^.

### Molecular dynamics simulation

Molecular dynamics simulations were conducted using GROMACS™, version 2022.3, to investigate the stability and behavior of the protein-ligand complex under dynamic conditions ^17^^,^^18^. Prior to simulation, the selected complex was prepared by separating the ligand from the protein structure using PyMOL™, version 2.5.3, followed by removal of crystallographic water molecules and other non-essential components.

Protein topology and coordinate files were generated within GROMACS employing the CHARMM36 force field, while ligand parameters were obtained using the CHARMM general force field ^19^. Subsequently, the protein-ligand complex was assembled, and the corresponding topology and coordinate files were generated.

The system was placed at the center of a cubic simulation box under periodic boundary conditions, maintaining a minimum solute-to-box distance of 1.5 nm to prevent interactions with periodic images. The simulation box was solvated using the SPC216 water model, resulting in approximately 17 230 water molecules, and appropriate counter-ions were added to neutralize the system charge.

Energy minimization was performed in two sequential stages using the steepest descent algorithm. Initially, position restraints were applied to both protein and ligand to allow solvent relaxation. This step was followed by an unrestrained minimization to reach a local minimum of the potential energy surface, with the maximum force threshold set to 100.0 kJ mol^1^ nm^-1^.

System equilibration was then achieved through two consecutive 100 ps equilibration phases: First, under constant volume and temperature (NVT ensemble), followed by constant pressure and temperature (NPT ensemble), stabilizing the system at 300 K and 1 bar.

Temperature control was maintained using the velocity-rescaling thermostat, while pressure coupling was applied using the Parrinello-Rahman method. The molecular dynamics simulation of the production run was carried out for 30 ns under the same thermodynamic conditions, employing a 2 fs integration time step and the leap-frog integrator. Short-range Lennard-Jones and Coulomb interactions were treated with a cutoff radius of 1.2 nm, and system coordinates were recorded every 10 ps for subsequent analysis.

Trajectory analysis was performed using visual molecular dynamics and Grace software, enabling evaluation of structural stability and dynamic behavior of the protein-ligand complex throughout the simulation.

## Results

### Effects of linalool on biofilm reduction

The effect of linalool on the biofilm formation of *C. albicans* strains LM 129 and ATCC 76485 was evaluated using the crystal violet staining method. A marked reduction in cell adhesion to the polystyrene surface was observed in both strains when compared to the negative control (table 1).

**Table 1.** Cell adhesion inhibition and corresponding classification observed in the *in vitro* biofilm assay

Drug	***Candida albicans* LM 129**		***Candida albicans* ATCC 76485**	
	Reduction (%) Adhesion tyoe		Reduction (%) Adhesion tyoe	
LIN	78,9 ± 2,3	Moderate	70,4 ± 1,9	Moderate
NYS	13,3 ±2,5	High	29,3 ± 2,5	High

LIN: linezolid; NYS: nystatin

* Data represent results from experiments performed in triplicate and are expressed as percentages of biofilm adhesion inhibition, calculated as mean ± SEM relative to the negative control.

The optical density measurements revealed inhibition percentages of 70.4 and 78.9% for the clinical and reference strains, respectively. According to the classification criteria described by Charleton ^13^ and Rodrigues *et al*. ^14^, antibiofilm activity is categorized as weak (<40%), moderate (60-80%), or strong (>80%) Therefore, the values obtained in this study are indicative of moderate antibiofilm activity.

In contrast, the antifungal agent nystatin showed only 13.3 and 29.3% inhibition, which corresponds to low antibiofilm efficacy and is classified within the high adhesion range under the same criteria. These findings reinforce the promising antibiofilm potential of linalool, particularly given its higher effectiveness relative to a standard antifungal agent. Furthermore, the consistent inhibition observed across both strains suggests that linalool’s activity is not strain-specific, indicating a possible broad-spectrum antibiofilm action.

Although statistical analyses such as t-tests or ANOVAwere not applied to this dataset due to the exploratory nature of the assay, the substantial inhibition percentages observed support the potential relevance of linalool in preventing *C. albicans* biofilm development.

### Interactions of linalool with 1,3-β-glucan synthase

**Figure 1.**
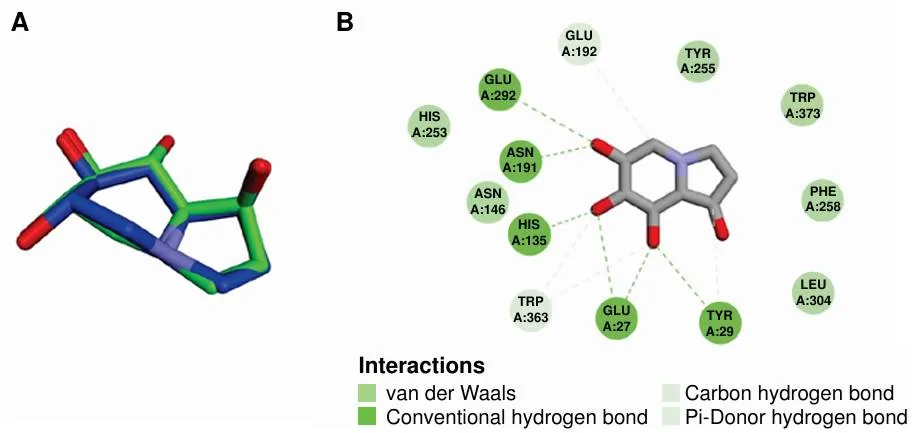
Docking protocol validation through castanospermine redocking. **A)** Structural overlap between the crystallographic castanospermine and the redocked pose, illustrating the accuracy of the docking procedure. **B)** Two-dimensional interaction map of castanospermine within the active site of *Candida albicans* 1,3-β-glucan synthase (Protein Data Bank ID: 1EQC), indicating hydrogen bonds, van der Waals contacts, Pi-donor hydrogen interactions, and C-H interactions.

ASN: asparagine; GLU: glutamic acid; HIS: histidine; LEU: leucine; PHE: phenylalanine; TRP: tryptophan; TYR: tyrosine

The docking protocol was validated through redocking of castanospermine, the co-crystallized ligand of *C. albicans* 1,3-β-glucan synthase (Protein Data Bank ID: 1EQC). The root-mean-square deviation (RMSD) value obtained (0.51 Å) was ≤2.0 Å, confirming the reliability of the docking method (figure 1A).

The binding free energy (*ΔG*) and inhibition constant (K_i_ ) for castanospermine were -7.0 kcal/mol and 0.41 µM, respectively, indicating strong affinity for the enzyme’s active site (table 2). Key molecular interactions included conventional hydrogen bonds with residues Glu27, Tyr29, His135, Asn191, and Glu292, as well as van der Waals, Pi-donor hydrogen bonds, and C-H interactions (figure 1B).

**Table 2:** Molecular docking parameters of linalool and castanospermine against *Candida albicans* 1,3-β-glucan synthase (Protein Data Bank ID: 1EQC).

Molecules	ΔG (kcal/mol)	RMSD (Å)	Ki
CAS	-7.0	0.51	0.41 pM
LIM	-6.0	-	39.85 pM

CAS: castanospermine; LIM: linalool; *ΔG*: Gibbs free energy change; RMSD: root mean square deviation; K: inhibition constant

**Figure 2.**
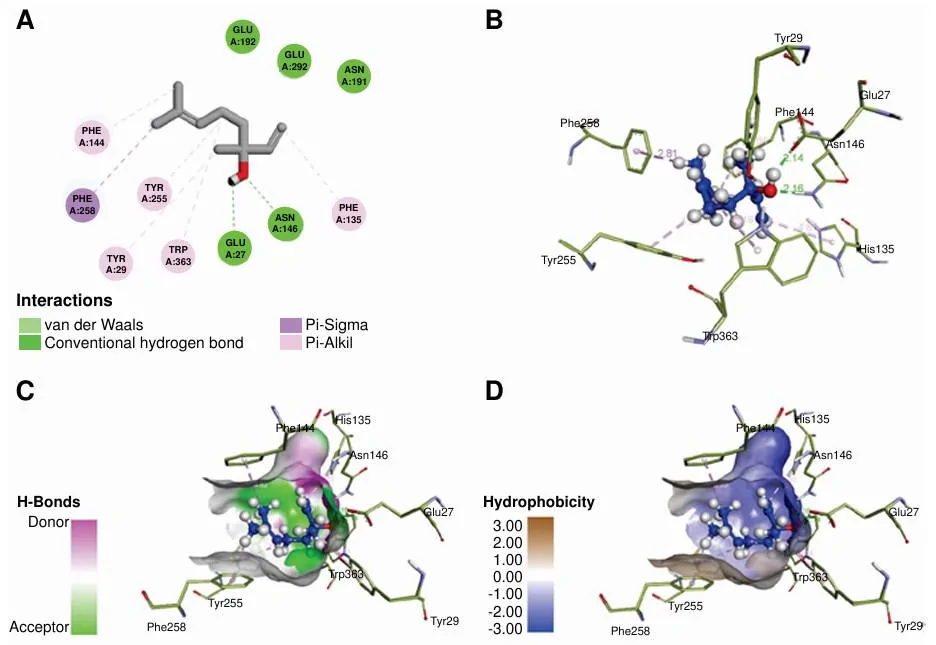
Binding mode of linalool within the active site of *Candida albicans* 1,3-β-glucan synthase (PDB ID: 1EQC). **A)** Two-dimensional representation of the interactions established between linalool and key catalytic residues. **B)** Three-dimensional depiction of the enzyme linalool complex highlighting ligand accommodation within the binding pocket. **C)** Surface representation of hydrogen bond donor and acceptor regions in the binding site. **D)** Hydrophobic surface mapping of the enzyme cavity involved in ligand recognition.

ASN: asparagine; GLU: glutamic acid; HIS: histidine; PHE: phenylalanine; TRP: tryptophan; TYR: tyrosine

Linalool, in turn, exhibited a binding energy of -6.0 kcal/mol and an inhibition constant of 39.85 µM, indicating lower affinity compared to castanospermine, but still within a potentially relevant inhibitory range (table 2). Molecular docking revealed that linalool established hydrogen bonds with Glu27 (2.14 Å) and Asn146 (2.16 Å), as well as a pi-sigma interaction with the aromatic ring of Phe258 (2.81 Å) (figures 2A and 2B). Additional van der Waals and Pi-alkyl interactions were also observed with residues within the enzyme’s catalytic cavity.

Importantly, these interactions occurred at the same active site occupied by castanospermine, supporting the hypothesis that linalool acts competitively on the enzyme, potentially inhibiting its activity by steric hindrance and disruption of critical catalytic interactions.

Additionally, the hydrogen bonding potential surface (figure 2C) and the hydrophobicity map (figure 2D) showed that, despite the predominantly polar nature of the binding cavity, the hydroxyl group of linalool allows for effective polar interactions with key residues. This dual character (polar and lipophilic) supports the interaction of linalool with the active site and reinforces its inhibitory potential.

### Molecular dynamics simulation calculations

Following the molecular docking analyses, the stability and dynamic behavior of the 1,3-β-glucan synthase-linalool complex were further investigated through 30 ns molecular dynamics simulation. This approach aimed to assess the persistence of ligand binding within the active site over time and to complement the static docking predictions with dynamic information regarding protein-ligand interactions.

**Figure 3.**
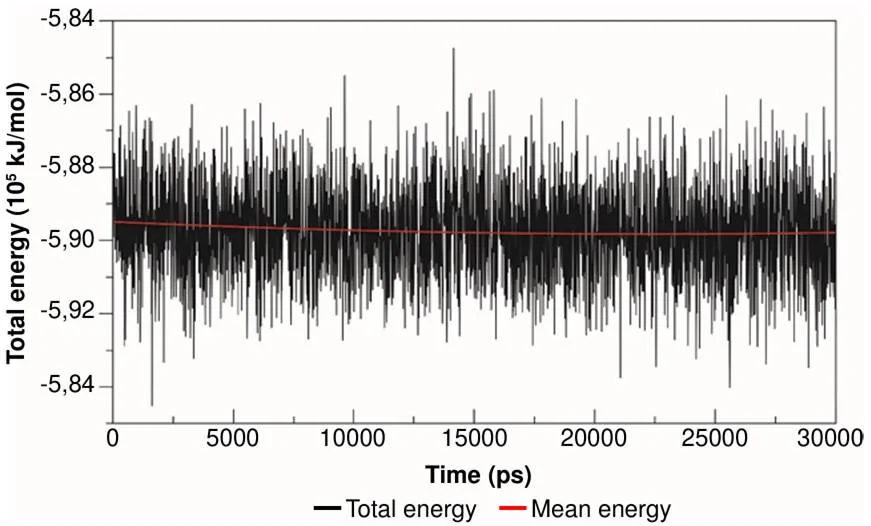
Total potential energy profile of the 1,3-β-glucan synthase-linalool complex during the molecular dynamics simulation. The temporal variation of the system’s total energy over the 30 ns simulation demonstrates convergence to a stable energetic state, with minor oscillations around the mean value (indicated by the red trend line).

The temporal evolution of the total potential energy of the system throughout the simulation is presented in figure 3. Although minor fluctuations were observed, the system rapidly reached an energetic plateau early in the simulation, with an average energy of approximately -5.905 ^x^ 10^5^ kJ/mol. The stabilization of the energy profile, highlighted by the trend line, indicates that the system attained a stable conformational state during the molecular dynamics run.

**Figure 4.**
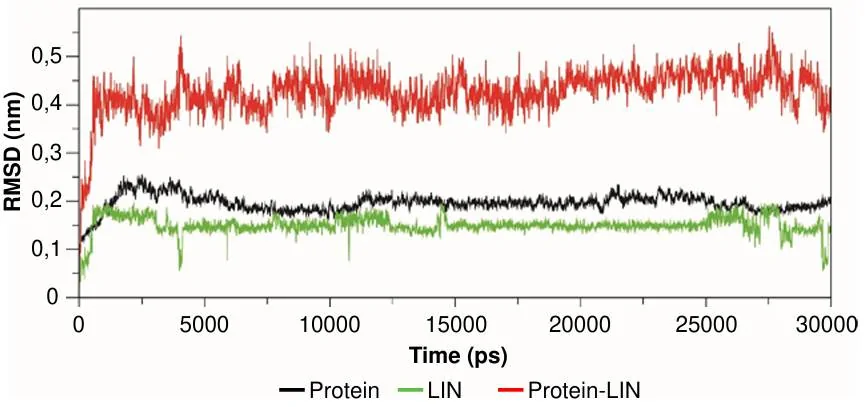
Root mean square deviation trajectories of the protein, linalool, and the protein-ligand complex during molecular dynamics simulation. The root mean square deviation profiles obtained over the 30 ns simulation period illustrate the conformational behavior and stability of the individual components and the assembled complex throughout the trajectory.

Structural stability of the protein, ligand, and protein-ligand complex was further evaluated by analyzing the RMSD values over the 30 ns trajectory (figure 4). After an initial adjustment phase, the RMSD deviation values for both the protein and the free ligand remained low and relatively constant. In contrast, the protein-ligand complex exhibited a moderate increase before reaching a new equilibrium state, still within a low deviation range. This behavior suggests conformational accommodation of the ligand within the active site while maintaining overall complex stability, corroborating the energy stabilization observed previously.

RMSD: root mean square deviation

**Figure 5.**
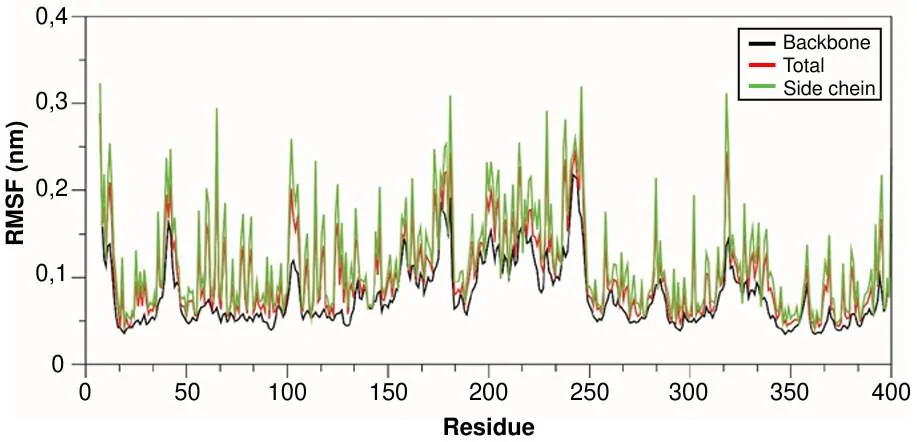
Residue-level flexibility of 1,3-β-glucan synthase during molecular dynamics simulation in the presence of linalool. RMSF values are shown for the entire protein, backbone atoms, and side chains, allowing identification of regions with increased conformational mobility along the amino acid sequence.

**Figure 6.**
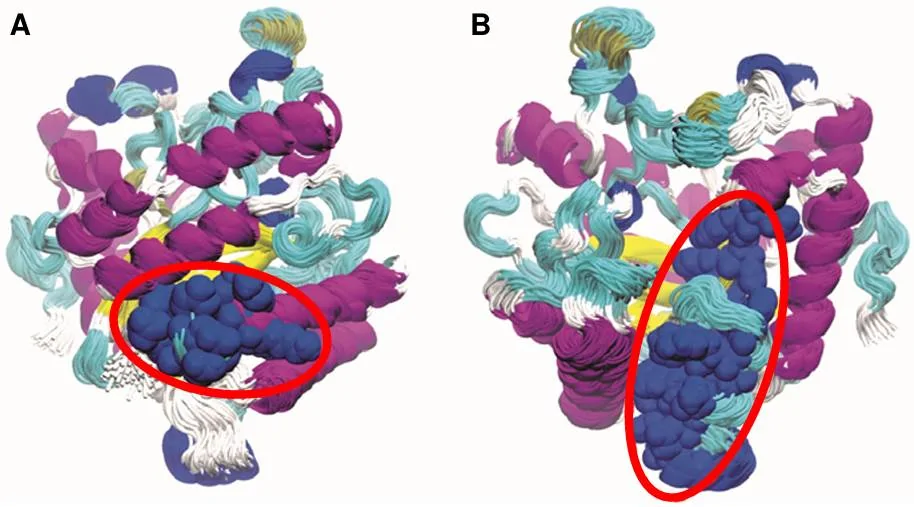
Structural mapping of highly flexible regions of 1,3-β-glucan synthase identified by RMSF analysis. A) Side-chain residues exhibiting elevated mobility located distal to the catalytic site. B) Highly flexible side-chain regions positioned near the entrance of the active site, indicating dynamic features that may influence ligand accommodation.

To investigate residue-level flexibility, root-mean-square fluctuation (RMSF) analyses were performed for the protein throughout the simulation (figure 5). Higher RMSF values in these analyses were predominantly associated with flexible regions such as side chains and loop segments, particularly within residues 172-188 and 232-256 (figures 6A and 6B). In contrast, residues located in the backbone region of the active site displayed reduced fluctuations, indicating greater structural rigidity. These findings support the notion that the binding site remained stable during the simulation and that linalool interacts moderately yet consistently with the enzyme, in agreement with docking results.

RMSF: root-mean-square fluctuatio

RMSF: root-mean-square fluctuation

**Figure 7.**
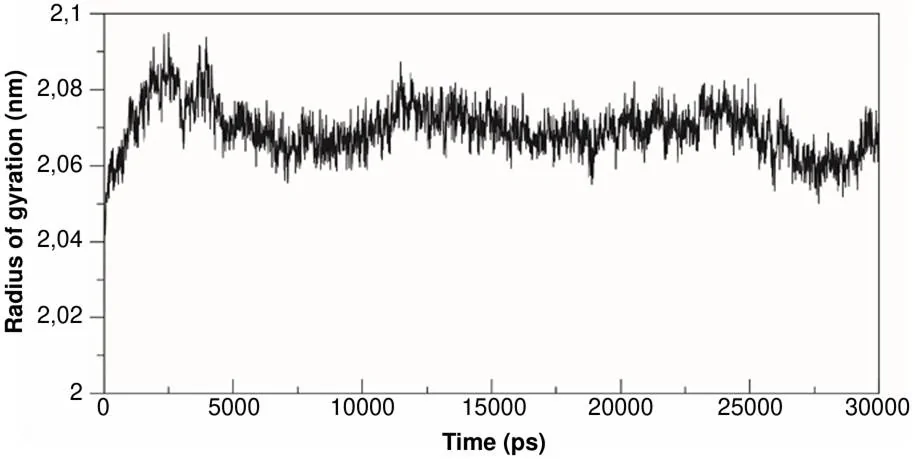
Radius of gyration profile of the 1,3-β-glucan synthase-linalool complex during molecular dynamics simulation. The Rg trajectory over the 30 ns simulation indicates maintenance of protein compactness, with only minor fluctuations observed throughout the simulation period.

The compactness of the protein-ligand complex was assessed through analysis of the radius of gyration over the simulation time (figure 7). An initial increase in values was observed, likely reflecting transient conformational adjustments of flexible regions. Subsequently, values stabilized, indicating maintenance of the overall structural compactness of the protein and a low tendency toward complex dissociation.

**Figure 8.**
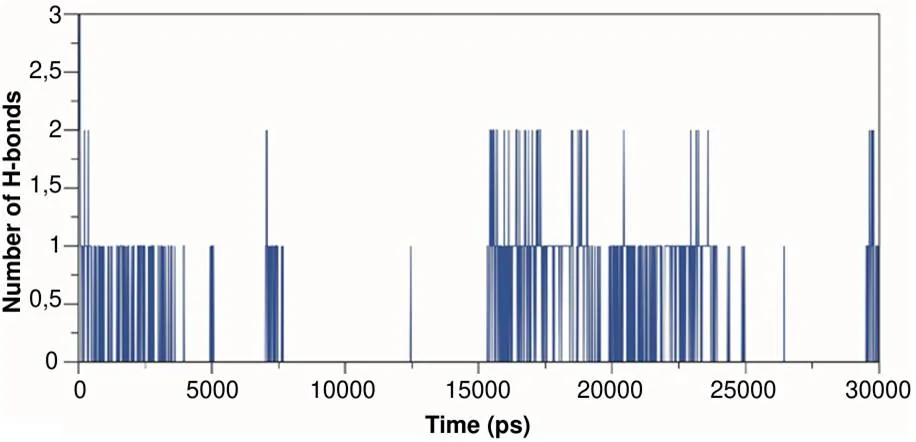
Temporal profile of hydrogen bond interactions between linalool and 1,3-β-glucano synthase during molecular dynamics simulation. The number of hydrogen bonds formed along the simulation trajectory reflects predominantly transient interactions, with intermittent hydrogen bond formation observed over time.

**Figure 9.**
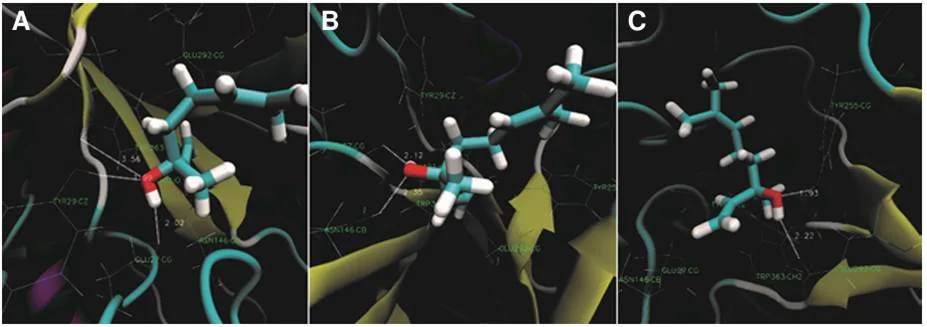
Three-dimensional snapshots illustrating hydrogen bond interactions between linalool and 1,3-β-glucan synthase at selected time intervals of the molecular dynamics simulation. A) Hydrogen bond interactions detected during the initial phase of the simulation (0-5 ns). B) Interactions observed during the intermediate interval (6.8-15 ns). C) Hydrogen bonding patterns identified during the later stage of the simulation (15.1-27.3 ns). Amino acid residues participating in the interactions are displayed in stick representation.

Hydrogen bond analyses were conducted to evaluate the persistence and dynamics of key ligand-protein interactions during the molecular dynamics simulation. At the initial stage of the simulation (0-5 ns), the hydroxyl group of linalool formed hydrogen bonds with active site residues Glu27 (2.02 Å), Tyr29 (1.99 Å), and Trp363 (3.56 Å), as illustrated in figures 8 and 9A.

Between approximately 6.8 and 15 ns, linalool established transient hydrogen bonds with Glu27 (2.12 Å) and Asn146 (2.35 Á) (figure 9B). These interactions were intermittently disrupted, reflecting the intrinsic flexibility of the ligand and surrounding residues, as evidenced by discontinuities in the hydrogen bond profile (figure 8).

During the interval from 15.1 to 27.3 ns, alternating hydrogen bond interactions were observed with Tyr255 (1.93 Å) and Glu292 (2.22 Å) (figure 9C). Toward the final stage of the simulation, hydrogen bonding interactions with Tyr29 and Trp363 were detected again.

Overall, the molecular dynamics simulation results demonstrate that linalool remains stably associated with the active site of 1,3-β-glucan synthase throughout the simulation, despite dynamic rearrangements of specific interactions. These findings reinforce the docking predictions and support the potential of linalool as a ligand capable of sustained interaction with the enzyme under dynamic conditions.

## Discussion

Vulvovaginal candidiasis remains a prevalent gynecological condition with a substantial clinical and socioeconomic impact, particularly due to its recurrent nature and the associated impairment in women’s quality of life ^1^. The recurrence of vulvovaginal candidiasis is widely recognized as a multifactorial process, influenced by host-related factors such as antibiotic exposure, metabolic disorders, hormonal variations during pregnancy, use of oral contraceptives, and conditions associated with immune dysfunction ^20^. Current therapeutic strategies are largely based on azole antifungal agents, as recommended by international clinical guidelines; however, the extensive and often repeated use of these drugs has contributed to the emergence of *C. albicans* strains with reduced susceptibility ^21^^,^^22^. This growing resistance scenario has been increasingly linked to therapeutic failure and persistent or recurrent infections, highlighting the need for alternative or complementary antifungal approaches ^23^.

Given the limitations of currently approved antifungal therapies -such as adverse effects and pharmacological interactions- there is a clear need for the development of alternative therapeutic agents that are both effective and safe. Natural product-derived compounds represent promising candidates due to their pharmacological versatility, relatively low toxicity, and demonstrated activity against resistant fungal strains ^24^^,^^25^.

Among the diverse classes of plant-derived compounds exhibiting antimicrobial potential, monoterpenes have attracted considerable attention due to their broad spectrum of biological activities. Linalool, a naturally occurring monoterpene commonly found in the essential oils of aromatic species such as *Lavandula angustifolia, Rosmarinus officinalis*, and *Ocimum basilicum*, has been extensively investigated for its antifungal properties *in vitro*.

In addition to its activity against fungal pathogens, linalool has been reported to exert anticonvulsant, anti-inflammatory, anxiolytic, antitumor, and antibacterial effects, supporting its relevance as a multifunctional bioactive molecule ^26^.

The ability of *C. albicans* to form biofilms is a major virulence factor and is frequently associated with antifungal resistance and infection recurrence ^4^. Biofilms are structured microbial communities embedded in a self-produced extracellular matrix that confers protection against antifungal agents and host immune responses.

Biofilm formation is promoted by factors such as the presence of medical devices, host immunosuppression, and prolonged antibiotic use. Consequently, biofilm-related pathogenesis should be taken into consideration during the diagnosis and treatment of vulvovaginal candidiasis to minimize recurrence ^5^.

In this context, the present study demonstrated that linalool exhibits antifungal activity by interfering with biofilm formation of *C. albicans* under *in vitro* conditions (table 1). On average, linalool reduced biofilm formation by 74.65% across both tested strains, and the level of cell adhesion to microtube walls was classified as moderate. In contrast, the standard antifungal nystatin exhibited a lower percentage of biofilm inhibition and a higher degree of cell adhesion. These findings are particularly relevant given that many commercial vaginal formulations for vulvovaginal candidiasis treatment contain nystatin, thus supporting the potential incorporation of linalool as an alternative or complementary agent ^27^^-^^29^.

Mechanistically, linalool appears to exert its antifungal effects by targeting 1,3-β-glucan synthase -an enzyme essential for the biosynthesis of the fungal cell wall- ^6^^,^^7^. In this study, molecular docking analyses indicated that linalool binds to the enzyme’s active site with a *ΔG* of -6.0 kcal/mol and an inhibitory constant (Ki) of 39.85 µM (table 2), demonstrating binding affinity.

As a lipophilic molecule, linalool can diffuse across cell membranes efficiently. Moreover, the partially apoiar character of the enzyme’s active site may facilitate favorable molecular interactions with linalool, enhancing ligand- target complementarity (figure 2).

Molecular dynamics simulations further supported these observations. The linalool-enzyme complex remained stable over a 30 ns simulation period, with no evidence of dissociation or significant structural deviation.

RMSD analysis confirmed structural stability throughout the simulation (figure 3A), while hydrogen bond analysis indicated persistent interactions - especially with residues Glu27 and Asnl 46- (figure 3B). These interactions suggest that linalool maintains a favorable binding orientation and contributes to the structural integrity of the complex under simulated physiological conditions.

Taken together, the docking and molecular dynamics results provide a coherent mechanistic explanation for the antifungal and antibiofilm effects observed *in vitro*. The integration of computational and experimental findings highlights linalool’s potential as an effective inhibitor of 1,3-β-glucan synthase and supports its candidacy as a promising natural compound for the treatment of *C. albicans*-associated vulvovaginal candidiasis.

Biofilm formation by *C. albicans* plays a central role in the persistence and recurrence of vulvovaginal candidiasis, representing a critical challenge for current antifungal therapies. In this scenario, targeting enzymes involved in cell wall biosynthesis, such as 1,3-β-glucan synthase, constitutes a rational strategy for antifungal intervention.

The results obtained in this study indicate that linalool can impair biofilm formation and establish stable interactions within the active site of 1,3-β-glucano synthase, as supported by molecular docking and molecular dynamics simulations. These findings highlight the potential of linalool as a bioactive natural compound with relevance for the development of alternative approaches against vulvovaginal candidiasis.

However, further experimental investigations, including comprehensive *in vitro* and *in vivo* studies, are necessary to confirm linalool’s antifungal effectiveness, assess safety parameters, and determine its applicability in clinical contexts.
